# Kaempferol induces autophagic cell death via IRE1-JNK-CHOP pathway and inhibition of G9a in gastric cancer cells

**DOI:** 10.1038/s41419-018-0930-1

**Published:** 2018-08-29

**Authors:** Tae Woo Kim, Seon Young Lee, Mia Kim, Chunhoo Cheon, Seong-Gyu Ko

**Affiliations:** 1 Department of Preventive Medicine, College of Korean Medicine, Kyung Hee University, Seoul, Korea; 2 Department of Cardiovascular and Neurologic disease (Stroke center), College of Korean Medicine, Kyung Hee University, Seoul, Korea

## Abstract

Kaempferol, a flavonoid, found in traditional medicine, fruits, and vegetables, and an HDAC inhibitor, is a powerful anti-cancer reagent against various cancer cell lines. However, detailed mechanisms involved in the treatment of gastric cancer (GC) using kaempferol are not fully understood. In our study, we investigated the biological activity and molecular mechanism involved in kaempferol-mediated treatment of GC. Kaempferol promoted autophagy and cell death, and increased LC3-I to LC3-II conversion and the downregulation of p62 in GC. Furthermore, our results showed that kaempferol induces autophagic cell death via the activation of the IRE1-JNK-CHOP signaling, indicating ER stress response. Indeed, the inhibition of ER stress suppressed kaempferol-induced autophagy and conferred prolonged cell survival, indicating autophagic cell death. We further showed that kaempferol mediates epigenetic change via the inhibition of G9a (HDAC/G9a axis) and also activates autophagic cell death. Taken together, our findings indicate that kaempferol activates the IRE1-JNK-CHOP signaling from cytosol to nucleus, and G9a inhibition activates autophagic cell death in GC cells.

## Introduction

Kaempferol is a natural flavonoid that is widely found in many fruits, vegetables, and traditional herbal medicine^1^. Kaempferol was recently reported to have anti-cancer properties against several cancers, including gastric, breast, lung, and renal cancer^2–5^. Flavonoids including kaempferol, quercetin, luteonin, and apigenin potentially function as HDAC inhibitors^6,7^. HDAC inhibitors induce cell death via diverse mechanisms, such as apoptosis, endoplasmic reticulum (ER) stress, autophagy, and epigenetic modification, and they have recently been suggested to be powerful cancer therapeutic agents^8–11^.

Research for anti-cancer effect by kaempferol indicates that it may inhibit the proliferation and expression of vascular endothelial growth factor (VEGF) in ovarian cancer cells^12^. Kaempferol induced cell cycle arrest and apoptosis via downregulation of cyclin B1, Cdk1, NF-κB and Bcl-2, and upregulation of Bax in HeLa cells and GC cells, implying that it has a therapeutic potential via anti-tumor effect^2,13^. On the basis of the reported molecular mechanisms, kaempferol, owing to its tumor-inhibiting properties, may be a potential chemotherapeutic strategy.

ER stress pathway is known as one of the apoptosis signaling in several diseases^14^. The sensors including pancreatic ER kinase (PERK), inositol-requiring-1 (IRE1), and activating transcription factor-6 (ATF6) are located in the ER membrane for stimulating ER stress^15^. Under ER stress response, PERK leads to eukaryotic translation initiation factor-2α (eIF2α) phosphorylation that causes induction of activating transcription factor-4 (ATF4) and -CCAAT-enhancer-binding protein homologous protein (CHOP)^16^. Active IRE1 removes a small intron from X-box-binding protein1 (XBP-1) mRNA and phosphorylates c-Jun N-terminal protein kinase-1 (JNK1)^16^. For instance, quercetin, a well-known flavonoid, induces cell death via activation of IRE1-JNK signaling and downregulation of Bcl-2 in colorectal cancer^17^. Apigenin causes cell death through PERK-eIF2α-ATF4-CHOP pathway in PC12 cells^18^. Caspase-12 is located in the ER and is activated during ER stress-induced cell death; however, caspase-12-deficient mice are resistant to ER stress-mediated cell death^19^. Recently, it has been demonstrated that a wide variety of flavonoids are able to regulate autophagic cell death via ER stress in many diseases^20^. Autophagy is a process wherein the cell digests cytoplasmic materials within lysosomes^21^. There are accumulating reports that autophagy has a dual role, including a tumor suppressive or promoting role^22^. Previous reports have demonstrated that ER stress-induced IRE1/JNK pathway results in Bcl-2/Beclin-1 inhibitory interactions leading to autophagy^23^. Beclin-1 is an important factor in autophagic cell death and interacts through its BH3 domain with anti-apoptotic Bcl-2^24^. The JNK1 mediates the  dissociation between Bcl-2/Beclin-1 complex and causes phosphorylation of Bcl-2^25^. Accumulating reports indicated that IRE1-mediated JNK activation is required for vacuole or autophagosome formation^26^. Autophagy is inhibited by the mammalian target of rapamycin (mTOR) and AMP-activated protein kinase (AMPK) binds to UNC-51-like kinase (ULK1), and this interaction contributes to autophagy activation^27,28^. The autophagy process is highly regulated by two kinases, ULK1 via AMPK/mTOR pathway and the class III phosphatidylinositol 3-kinse (VPS34) by regulating FIP200, Beclin-1, and autophagy-related (ATG) proteins^29^. From microtubule-associated protein light chain 3 I (LC3-I) to LC3-II translocated to the autophagosome membrane and it formed autolysosome by fusing with lysosomes and subsequently degraded^30^.

Emerging reports have indicated that many flavonoids mediate autophagy in cancer and that kaempferol mediates autophagy via AMPK/mTOR signaling in cancer cells^31^. Recent reports suggest that inhibition of histone methyltransferase, including G9a, induces autophagy via AMPK/mTOR pathway^32^. For example, depsipeptide, an HDAC inhibitor, decreases H3K9me2 expression via inhibition of G9a^33^. A previous report found that G9a was upregulated in human cancers and that G9a knockdown inhibited cell growth and metastasis by inducing apoptosis and autophagy^34^. G9a inhibition-mediated autophagic cell death was regulated by mTOR/AMPK/ULK1 axis^35^. Furthermore, inhibition of HDAC/G9a pathway has anti-tumor effect and may have a critical role in the chemotherapeutic efficacy of cancer^36^. Epigenetic compounds, including HDAC and DNMT inhibitors, are used for more effective cancer treatment strategies in conjunction with various chemotherapies^37^. However, detailed research on whether kaempferol regulates autophagic cell death via epigenetic modification in GC is still not clear.

In the present study, we sought to examine whether kaempferol induces autophagic cell death via ER stress and epigenetic modification in GC. We demonstrate that kaempferol induces autophagic cell death via IRE1-JNK1 axis and HDAC/G9a pathway in GC, thus broadening our understanding for kaempferol as an anti-cancer agent.

## Results

### Effects of kaempferol in GC cell lines

**Fig. 1:**
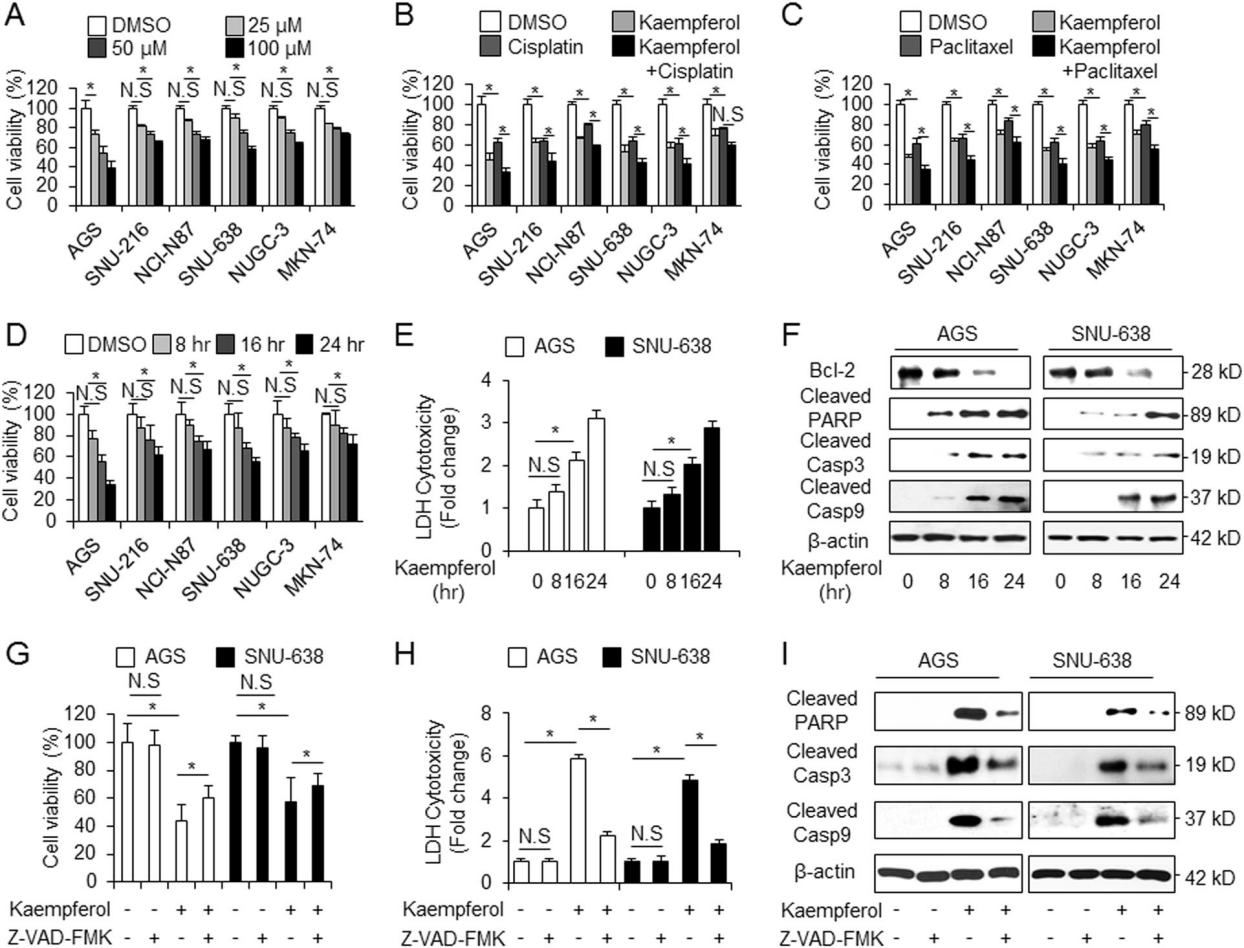
Cytotoxic effects of kaempferol in GC cell lines. **a**–**d** Cell viability of kaempferol, cisplatin, and paclitaxel in GC cell lines, including AGS, SNU-216, NCI-N87, SNU-638. NUGC-3 and MKN-74, measured using WST-1 on 96-well plates. **a**, **d** Kaempferol is treated in a dose-dependent manner (25 μM, 50 μM, and 100 μM; 24 h) and a time-dependent manner (8, 16, and 24 h). **b**, **c** Kaempferol-treated GC cells were treated with cisplatin (5 µM, 24 h, Sigma) or paclitaxel (50 nM, 24 h, Sigma). Cell viability of DMSO-treated control cells was set at 100%; *p *<* 0.05. **e, f** LDH activity in a time-dependent manner (8, 16, 24 h) in kaempferol (50 μM)-treated AGS and SNU-638 cells. Western blotting of cleaved caspase-3, -9, cleaved PARP and Bcl-2 analyzed for the indicated times in kaempferol-treated AGS and SNU-638 cells; *p *<* 0.05. **g**–**i** Effect of Z-VAD-FMK (50 μM) on kaempferol-induced cell death. AGS and SNU-638 cells were pretreated with Z-VAD-FMK for 4 h and were subsequently treated with kaempferol (50 μM, 24 h). Cell viability was determined using the WST-1 assay, and cell cytotoxicity was monitored using LDH assay; *p *<* 0.05. Sampling of total lysates was conducted by western blot assay to identify the activation of apoptosis markers such as cleaved caspase-3, -9, and cleaved PARP. β-actin was used as a protein loading control

To investigate the cytotoxic effect of kaempferol in GC, we measured the changes of cell viability by kaempferol using WST-1 assay on indicated concentration (Fig. 1a). Kaempferol causes a significant decrease of cell viability compared with DMSO (Fig. 1a). Next, we examined the effect of cisplatin (5 µM, 24 h) or paclitaxel (50 nM, 24 h) in combination with kaempferol (Fig. 1b, c). GC cells treated with cisplatin or paclitaxel in combination with kaempferol showed lower cell viability than those treated with only cisplatin or paclitaxel. To investigate the time-dependent effects of kaempferol, kaempferol treated in GC cells in indicated times (Fig. 1d). Time course experiments showed that kaempferol (50 μM) decreased cell viability in GC cells compared with control. These findings suggest that kaempferol significantly decreases cell viability in GC cells and cisplatin or paclitaxel in combination with kaempferol have a powerful multi-drug cytotoxic effect on GC cells.

### Kaempferol induces cell death in GC cells

We examined kaempferol’s biological effect to identify whether this induces cell death in GC cells. After the cells were exposed to kaempferol (50 μM) at indicated time points, cell death rate was determined by LDH assay (Fig. 1e). The data showed that kaempferol induces ~3 times higher LDH release than DMSO in a time-dependent manner. Western blotting demonstrated that kaempferol (50 μM) increased cleaved caspase-3 and -9 and reduced Bcl-2 levels to a greater extent than DMSO in a time-dependent manner (Fig. 1f). To better characterize whether the kaempferol-treated cell death is apoptotic, we treated the cells pretreated with pan-caspase inhibitor, Z-VAD-FMK (50 µM) for 4 h, with kaempferol (Fig. 1g). Consequently, Z-VAD-FMK + kaempferol sufficiently inhibited cell viability and LDH release compared to kaempferol alone (Fig. 1g, h). Western blotting demonstrates that Z-VAD-FMK + kaempferol decreased cleaved caspase-3 and -9 and cleaved PARP to a greater extent than kaempferol alone (Fig. 1i). Taken together, these findings suggest that kaempferol induces caspase-dependent cell death in GC.

### Kaempferol induces autophagy in GC cells

**Fig. 2:**
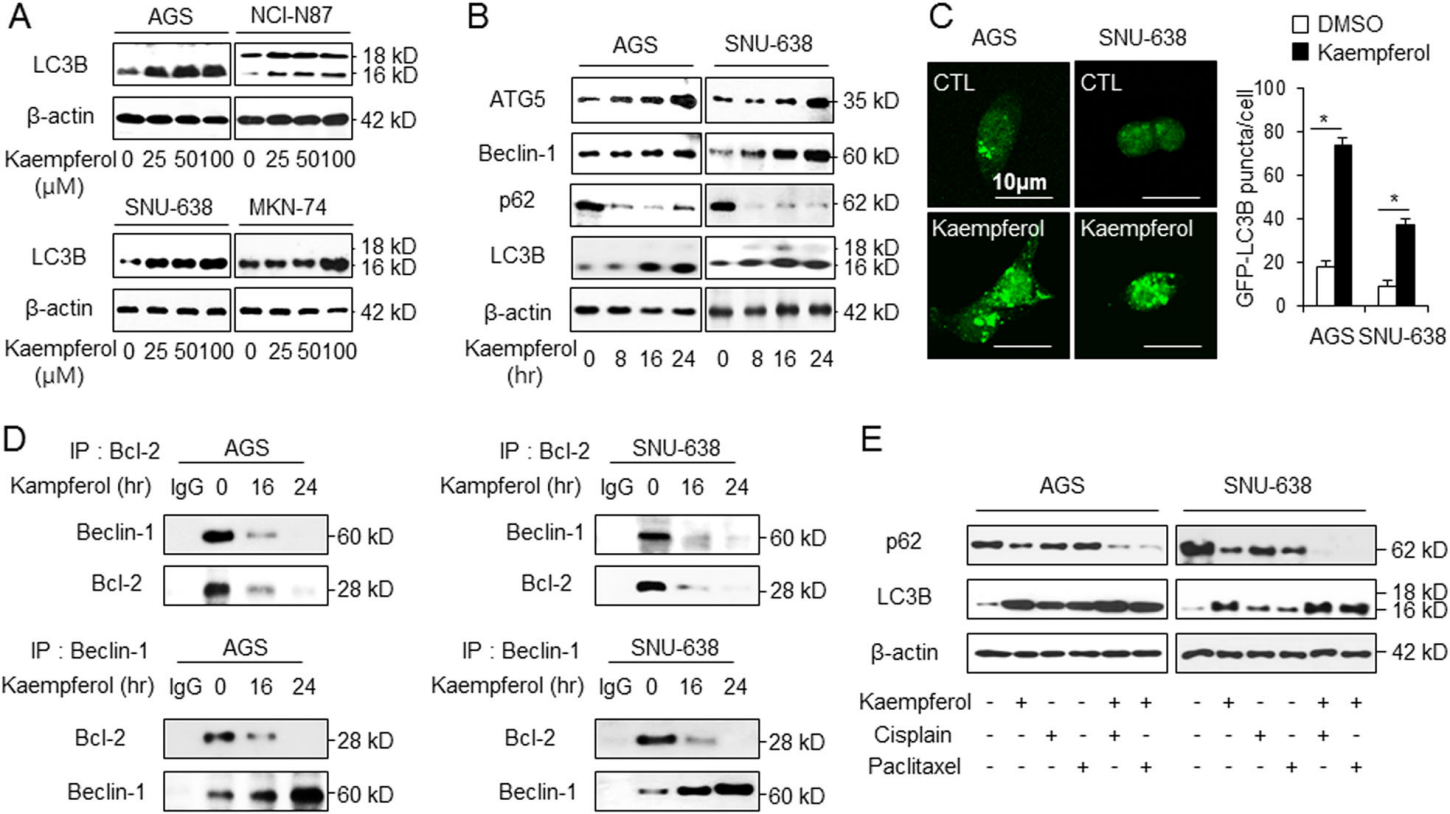
Induction of autophagy in kaempferol-treated GC cell lines. **a** Western blot analysis of microtubule-associated protein 1 A/1B-light chain 3 (LC3)-I to LC3-II conversion in GC cells including AGS, NCI-N87, SNU-638, and MKN-74. **b** Western blot analysis of ATG5, Beclin-1, p62, and LC3B protein levels in AGS and SNU-638 cells treated with kaempferol (50 μM) for the indicated times. **c** GC, AGS, and SNU-638 cells transfected using the pEGFP-LC3 vector were treated with kaempferol (50 μM) for 8 h and analyzed as described in the material and methods. Fluorescence microscopy analysis calculated by punctate of LC3B staining; *p *<* 0.05. **d** AGS cells were treated with kaempferol (50 μM) for indicated times. Bcl-2 was immunoprecipitated in AGS and SNU-638 cells, and the immunoprecipitated proteins were subjected to western blotting. Beclin-1 was detected in immunoprecipitates prepared with anti-Bcl-2 antibody by immunoprecipitation. Bcl-2 was also monitored in immunoprecipitates prepared with anti-Beclin-1 antibody by immumoprecipitation. **e** After AGS and SNU-638 cells were treated with cisplatin (5 μM) or paclitaxel (50 nM) in combination with kaempferol (50 µM) for 24 h, protein samples were loaded to Western blotting for LC3B and p62. β-actin was used as a protein loading control

We determined whether or not kaempferol induces autophagy in GC cells. Western blotting was performed to examine the expression of the autophagy marker LC3B. Unlike with DMSO treatment, with kamepferol treatment, LC3-II expression in GC cells showed dose-dependent increase (Fig. 2a). To further investigate whether kaempferol mediates autophagy in GC cells, we monitored time-dependent expression of autophagy-related proteins including LC3B, p62, Beclin-1, and ATG5 in kaempferol-treated GC cells. We identified that kaempferol increases the expression of LC3-II, Beclin-1, and ATG-5 and decreases p62 levels to a greater extent than DMSO treatment (Fig. 2b). Furthermore, green fluorescent protein (GFP)-LC3 plasmid was transfected into GC cells to monitor kaempferol-induced autophagy. The formation of GFP-LC3B puncta indicates the formation of autophagosomes^38^. Kaempferol induced ~4-fold and 2-fold pronounced formation of LC3 puncta in GC cells compared with DMSO (Fig. 2c). A previous report suggests that AMPKα activation stimulates autophagy via the dissociation of the Beclin-1–Bcl-2 complex^39^. To investigate the dissociation of Beclin-1–Bcl-2 complex in kaempferol-treated GC cells, co-immunoprecipitation (IP) using antibodies for Beclin-1 and Bcl-2 was performed (Fig. 2d). We found that the interaction between Beclin-1 and Bcl-2 was weaker shown to be weaker in kaempferol-treated cells than in DMSO-treated cells. To check whether a combination of cisplatin (5 µM) or paclitaxel (50 nM) with kaempferol (50 µM) regulates autophagy, we checked the expression of autophagy markers using Western blotting (Fig. 2e). Kaempferol-treated cells had higher expression of LC3B than cisplatin- or paclitaxel-treated cells. However, they had lower expression of p62 than cisplatin- or paclitaxel-treated cells. Moreover, cisplatin + kaempferol and paclitaxel + kaempferol treatments induce more autophagy than kaempferol alone. Taken together, our findings suggest that cisplatin or paclitaxel in combination with kaempferol regulated cell death via autophagy activation in GC.

### Kaempferol regulates autophagic flux in GC cells

**Fig. 3:**
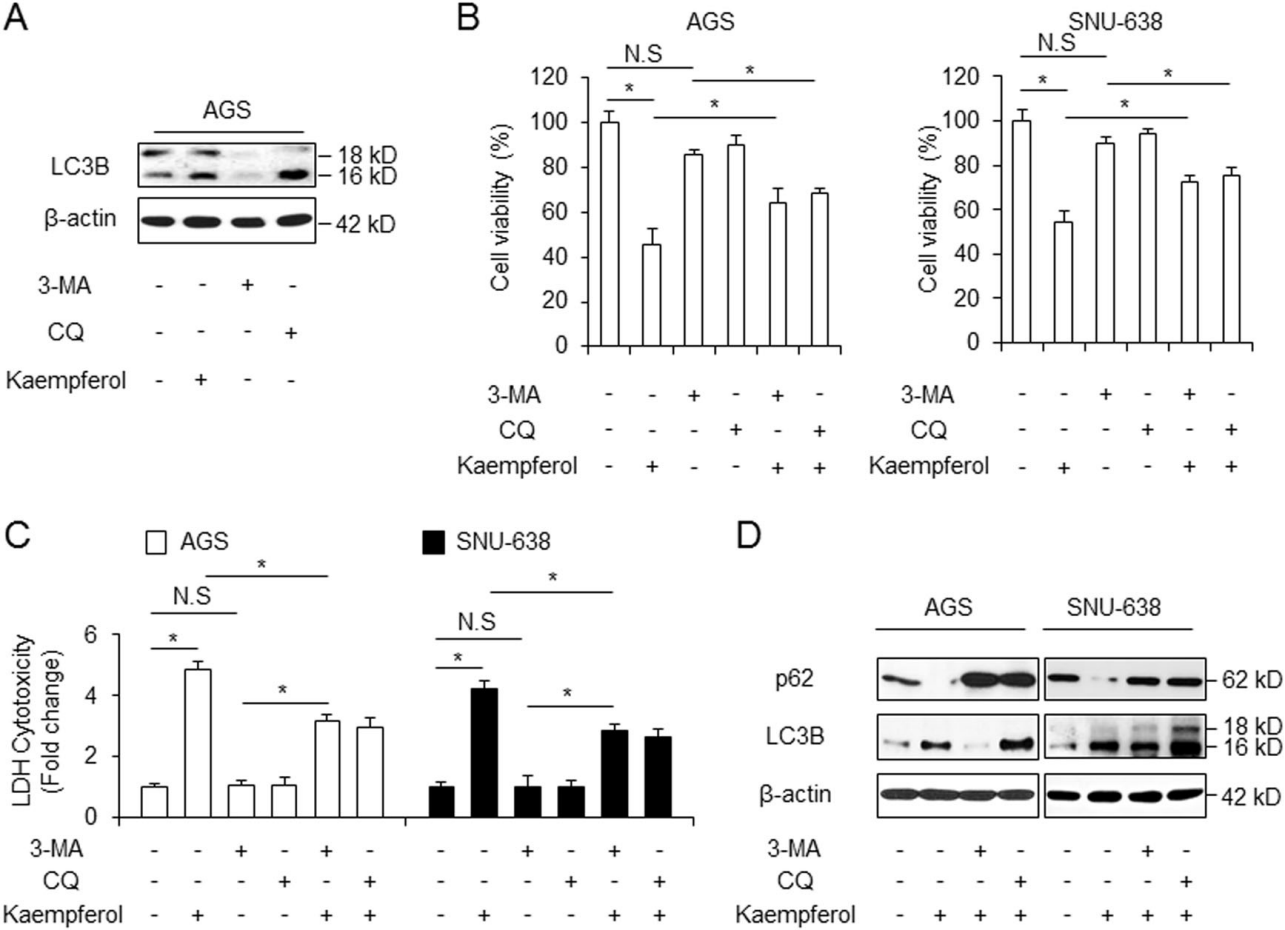
Regulation of autophagy flux in kaempferol-treated GC cells. **a** Western blot analysis of LC3B in AGS cells treated with DMSO or autophagy inhibitor, such as 3-MA (5 mM) and CQ (20 μM) for 24 h. **b,** **c** Cell viability was analyzed using WST-1 and LDH release in kaempferol-treated AGS and SNU-638 cells with 3-MA (5 mM) or CQ (20 μM) treatment; *p *<* 0.05. **d** Western blot analysis of LC3B and p62 in the kaempferol-treated AGS and SNU-638 cells with 3-MA (5 mM) or CQ (20 μM)

To confirm the biological role of autophagy in kaempferol-treated GC cells, we performed autophagic flux experiment using autophagy inhibitor, 3-methyladenine (3-MA) and chloroquine (CQ). The class III PI3K inhibitor 3-MA is an early-stage autophagy inhibitor that blocks autophagosome formation, whereas CQ prevents the fusion of autophagosomes with lysosomes and inhibits the lysosomal degradation of proteins at the late stage of autophagy^40^. Autophagic flux experiment by 3-MA (5 mM) and CQ (20 µM) was verified by Western blotting (Fig. 3a). Unlike kaempferol, 3-MA reduced the expression of LC3-II, indicating the blockage of autophagosome formation. Further, unlike kaempferol, CQ accumulated the expression of LC3-II, indicating prevention of the fusion of autophagosomes with lysosomes (Fig. 3a). To investigate whether kaempferol in combination with autophagy inhibitors regulates autophagic flux, kaempferol (50 µM) was exposed after GC cells were pretreated with 3-MA (5 mM) or CQ (20 µM) for 4 h. Our data indicate that 3-MA and CQ did not affect cell viability and LDH release, but kaempferol + autophagy inhibitors dramatically enhanced cell viability and reduced LDH release to a greater extent than kaempferol alone did (Fig. 3b, c). These results suggest that kaempferol + autophagy inhibitors inhibit cell death to a greater extent than kaempferol alone in GC cells. We checked LC3B and p62 levels using western blotting. When GC cells were treated with kaempferol+ 3-MA, p62 increased and LC3-II decreased, compared with DMSO (Fig. 3d). Furthermore, kaempferol+ CQ increased p62 and LC3-II levels, indicating that kaempferol regulates autophagic flux (Fig. 3d). Therefore, our evidence indicates that kaempferol regulates autophagic flux in GC.

### Autophagy inhibition suppresses kaempferol-induced cell death in GC cells

**Fig. 4:**
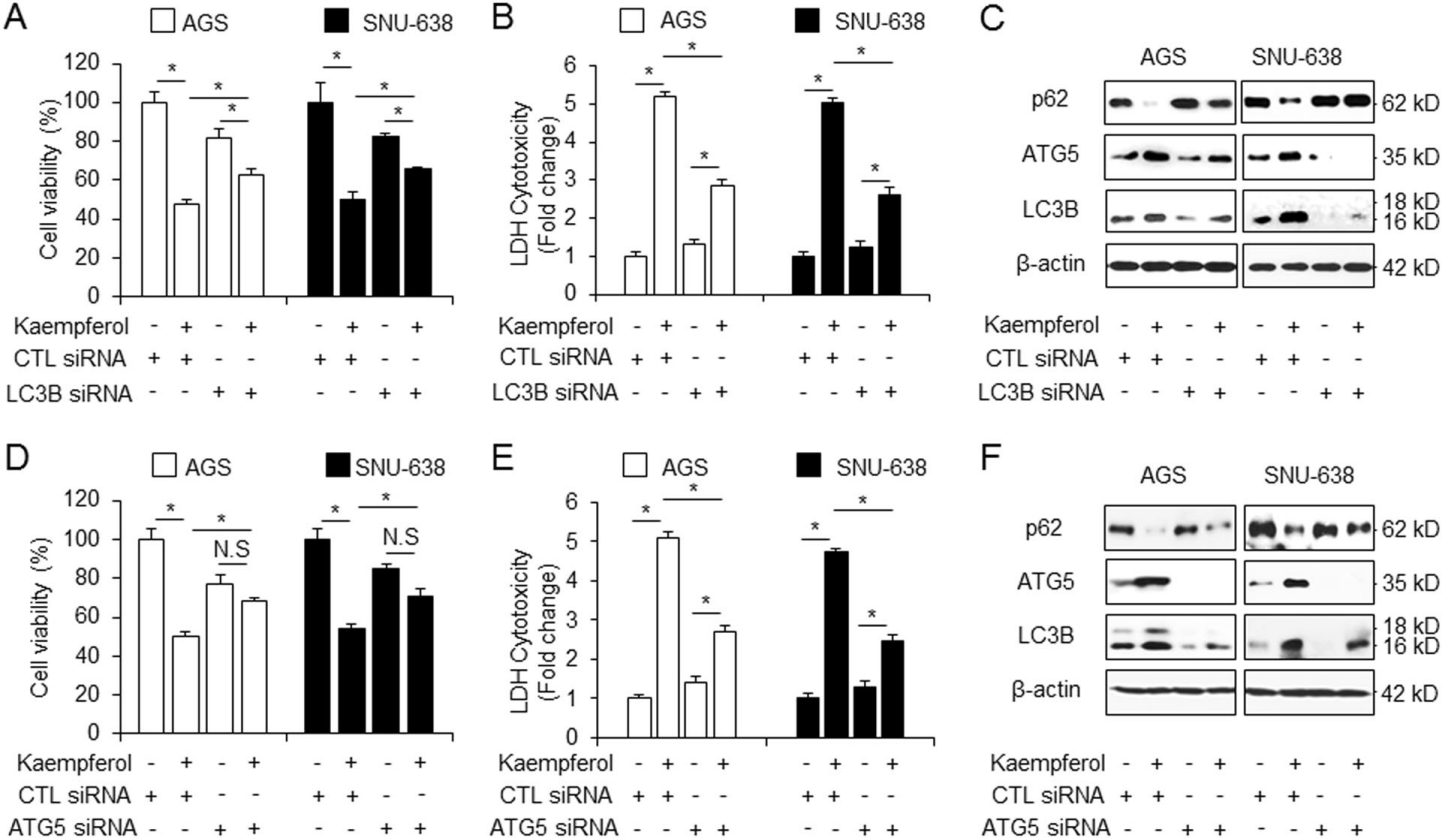
Kaempferol-stimulated cell survival via autophagy inhibition. **a**–**f** After AGS and SNU-638 cells were transfected with LC3B and ATG5 siRNA, cell viability and LDH activity analyses and Western blot assay were performed with/without kaempferol (50 μM, 24 h) treatment. **a**, **b**, **d**, **e** Cell viability and LDH activity were determined using WST-1 and LDH assay, respectively; *p *<* 0.05. **c**, **f** Western blotting was performed to identify the autophagy-related genes, such as p62, ATG5, and LC3B, in kaempferol- treated LC3B knockdown cells siRNA. β-actin was used as a protein loading control

To further confirm whether autophagy inhibition regulates kaempferol-induced cell death or not, knockdown experiments, including LC3B and ATG5 siRNA, were performed to identify kaempferol-induced autophagic cell death. Our data showed that compared with the control siRNA+ kaempferol treatment, GC cells transfected with LC3B and ATG5 siRNA exhibited better cell viability and lower levels of LDH release in kaempferol-treated cells (Fig. 4a, b, d, e). LC3B and ATG5 knockdown attenuated LC3B and ATG5 levels and increased p62 levels in kaempferol-treated GC cells to a great extent than the control siRNA+ kaempferol treatment did (Fig. 4c). Our findings suggest that autophagy inhibition blocks cell death in kaempferol-induced GC.

### AMPK/ULK1 regulates kaempferol-induced autophagic cell death in GC cells

**Fig. 5:**
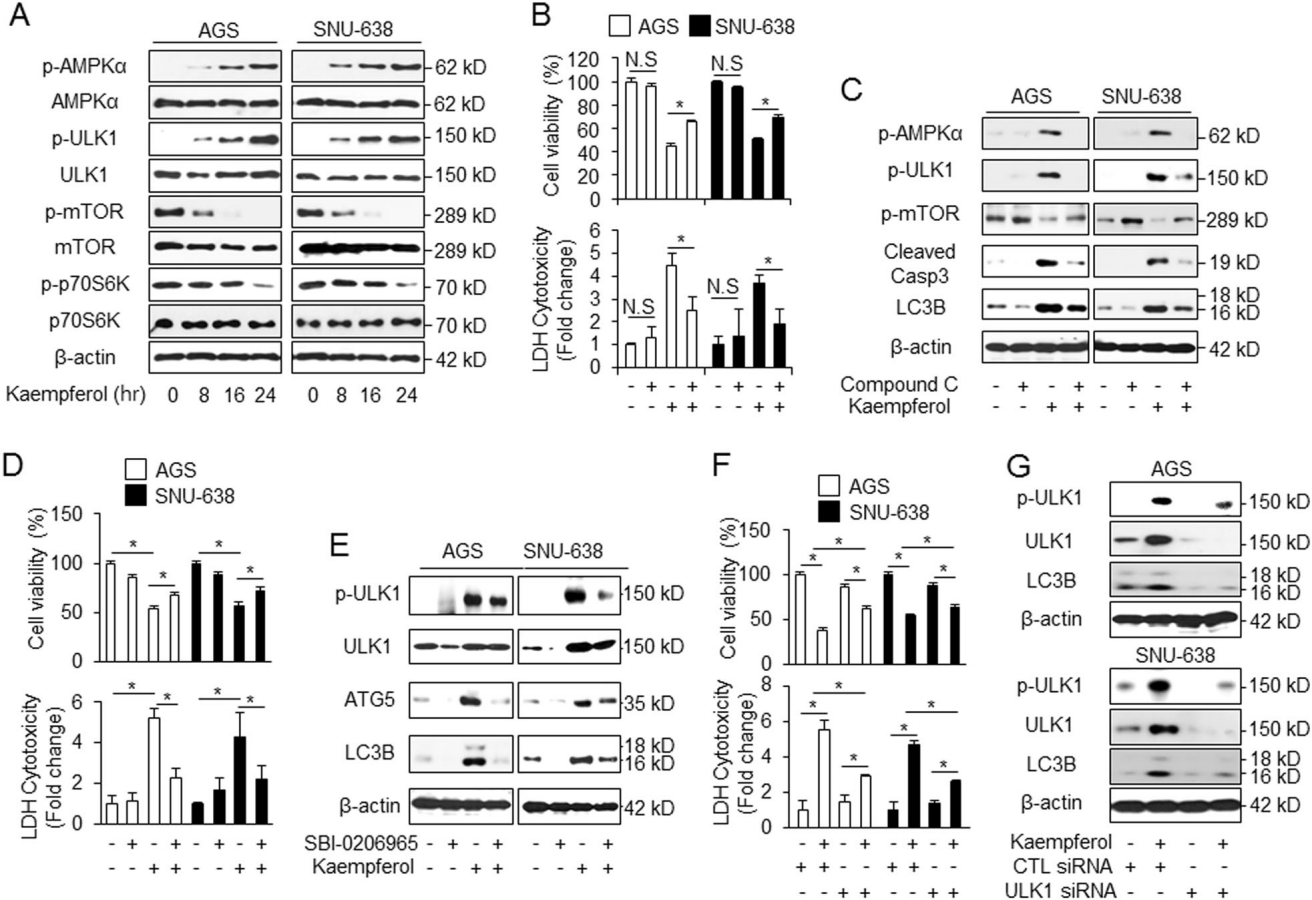
Inhibition of AMPKα/ULK1 attenuates cell death in kaempferol-treated GC cells. **a** AGS and SNU-638 cells were treated with kaempferol (50 μM) in a time-dependent manner (0, 8, 16, and 24 h). Cell lysates were loaded used in the western blot assay and detected antibodies targeting p-AMPKα (Thr172), AMPKα, p-ULK1 (Ser555), ULK1, p-mTOR (Ser2448), mTOR, p-p70S6K (Thr389), and p70S6K involved in autophagy induction. β-actin was used as a protein loading control. **b**, **c** The effect of cell viability, LDH release, and Western blot analysis for p-AMPKα, p-ULK1, p-mTOR, cleaved caspase-3, and LC3B in kaempferol (50 μM, 24 h)-treated AGS and SNU-638 cells in the presence or absence of compound C (2 μM, 24 h); *p < 0.05. **d**, **e** The effect of cell viability, LDH release, and western blot analysis for LC3B, ATG5, p-ULK1, and ULK1 in kaempferol (50 μM, 24 h)-treated AGS and SNU-638 cells in the presence or absence of SBI-0206965 (10 μM, 24 h); *p < 0.05. **f**, **g** Cell viability, LDH release, and western blot analysis of LC3B, p-ULK1, and ULK1 in AGS and SNU-638 cells treated with kaempferol (50 μM, 24 h) in the presence or absence of ULK1 siRNA (30 nM, 24 h); *p *<* 0.05

Using western blotting, we investigated whether the AMPKα/ULK1 and mTOR/p70S6K pathways were involved in kaempferol-induced autophagy in GC cells. Kaempferol induced a time-dependent decrease in the p-mTOR, but did not affect total mTOR levels (Fig. 5a). Moreover, kaempferol mediated a reduction in the p-p70S6K (Fig. 5a). We examined the activation of AMPKα and ULK1 in kaempferol-treated GC cells. AMPKα and ULK1 could be activated by kaempferol in a time-dependent manner (Fig. 5a). As expected, compound C (2 µM), a well-known inhibitor of AMPK, induced kaempferol-mediated cell survival, which was indicated by a greater increase in the cell viability and a decrease in the LDH release compared with kaempferol alone (Fig. 5b). Compound C resulted in lower expression of p-AMPKα, p-ULK1, cleaved caspase-3, and LC3 lipidation in the kaempferol-treated GC cells, compared to that observed with kaempferol alone (Fig. 5c). These findings indicated that the AMPKα inhibition interrupted kaempferol-mediated autophagic cell death and promoted cell survival (Fig. 5c). To examine how ULK1 regulates kaempferol-induced autophagy, we investigated whether kaempferol-induced autophagy was regulated by SBI-0206965, a ULK1 inhibitor. SBI-0206965 (10 µM) + kaempferol (50 µM) slightly increased cell viability compared to kaempferol alone (Fig. 5d). Furthermore, we found that kaempferol induces the expression of ULK1, ATG5, and LC3B, whereas kaempferol+ SBI-0206965 strongly decreased LC3B, ATG5, and ULK1 levels (Fig. 5e). To further investigate the role of ULK1 on kaempferol-induced autophagy, we performed loss-of-function study using ULK1 siRNA. Our findings consistently showed that the inhibition of ULK1 with kaempferol led to cell survival compared with kaempferol-treated control cells and that the kaempferol-induced LC3-II levels were significantly reduced in ULK1 knockdowned cells compared with control cells (Fig. 5f, g). Therefore, our data indicated that kaempferol induces autophagic cell death via AMPK-ULK1 pathways in GC.

### Kaempferol induces autophagic cell death via IRE1 signaling in GC cells

**Fig. 6:**
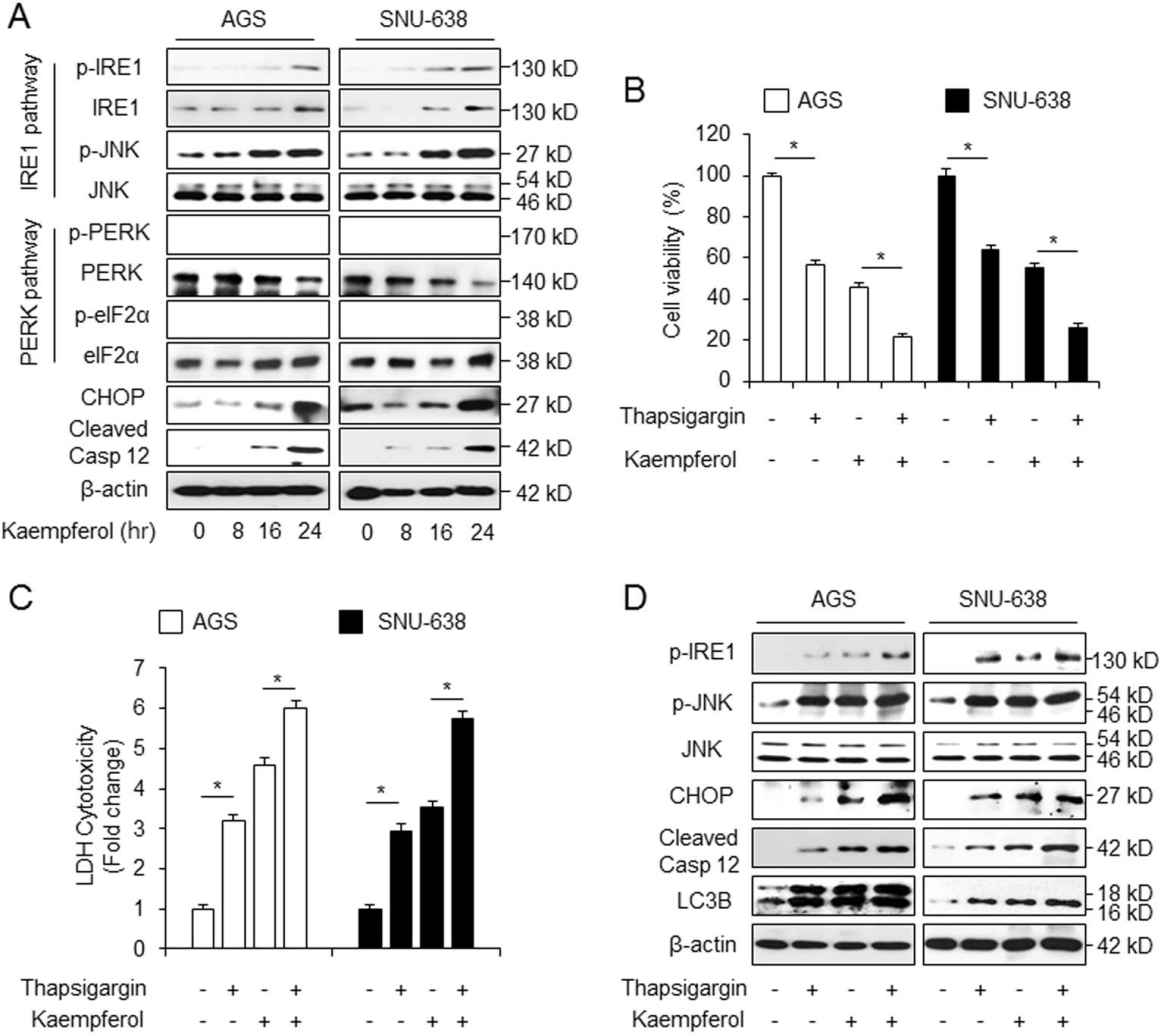
Kaempferol induces cell death and autophagy via ER stress pathway. **a** AGS and SNU-638 cells were treated with kaempferol (50 μM) for indicated times, and the activation of ER stress signaling, including p-IRE1, IRE1, p-JNK, JNK, p-PERK, PERK, p-eIF2α, eIF2α, CHOP, and cleaved caspase-12 was assessed using western blot assay. **b**–**d** AGS and SNU-638 cells were treated with thapsigargin (3 μM, 24 h) and kaempferol (50 μM, 24 h), and cell viability and LDH release were determined using WST-1 and LDH assays, respectively; *p *<* 0.05. **d** IRE1 signaling, including p-IRE1, p-JNK, JNK, CHOP, cleaved caspase-12, and LC3B were evaluated by Western blotting. β-actin was used as a protein loading control

Recent report indicates that ER stress is associated with autophagy and apoptosis pathway^41^. To determine whether kaempferol induces ER stress, we examined the expression of ER stress markers including IRE1 and PERK. In the present study, we found that the p-IRE1 levels show a time-dependent increase in response to kaempferol treatment (Fig. 6a). Kaempferol also stimulated p-JNK and CHOP, indicating the activation of IRE1 pathway (Fig. 6a). However, PERK signaling was not detected in kaempferol-treated cells (Fig. 6a). These results suggest that kaempferol induces ER stress via IRE1 signaling in GC. To evaluate whether thapsigargin (ER stress inducer, 3 µM) in combination with kaempferol (50 µM) activates ER stress signaling in GC cells, we examined cell viability and LDH release. Our results indicated that unlike DMSO, kaempferol or thapsigargin alone reduced cell viability and increased LDH release. Interestingly, kaempferol + thapsigargin decreased cell viability and enhanced LDH release to a greater extent compared with kaempferol or thapsigargin alone (Fig. 6b, c). Next, we examined the expression of the ER stress markers in thapsigargin and kaempferol-treated GC cells. Kaempferol and thapsigargin exhibited increased expression of p-IRE1, p-JNK, CHOP, cleaved caspase-12, and LC3-II, indicating activation of IRE1 pathway (Fig. 6d). Therefore, this indicates that kaempferol induces autophagic cell death via IRE1 signaling in GC.

### IRE1 inhibition suppresses kaempferol-induced autophagic cell death in GC cells

**Fig. 7:**
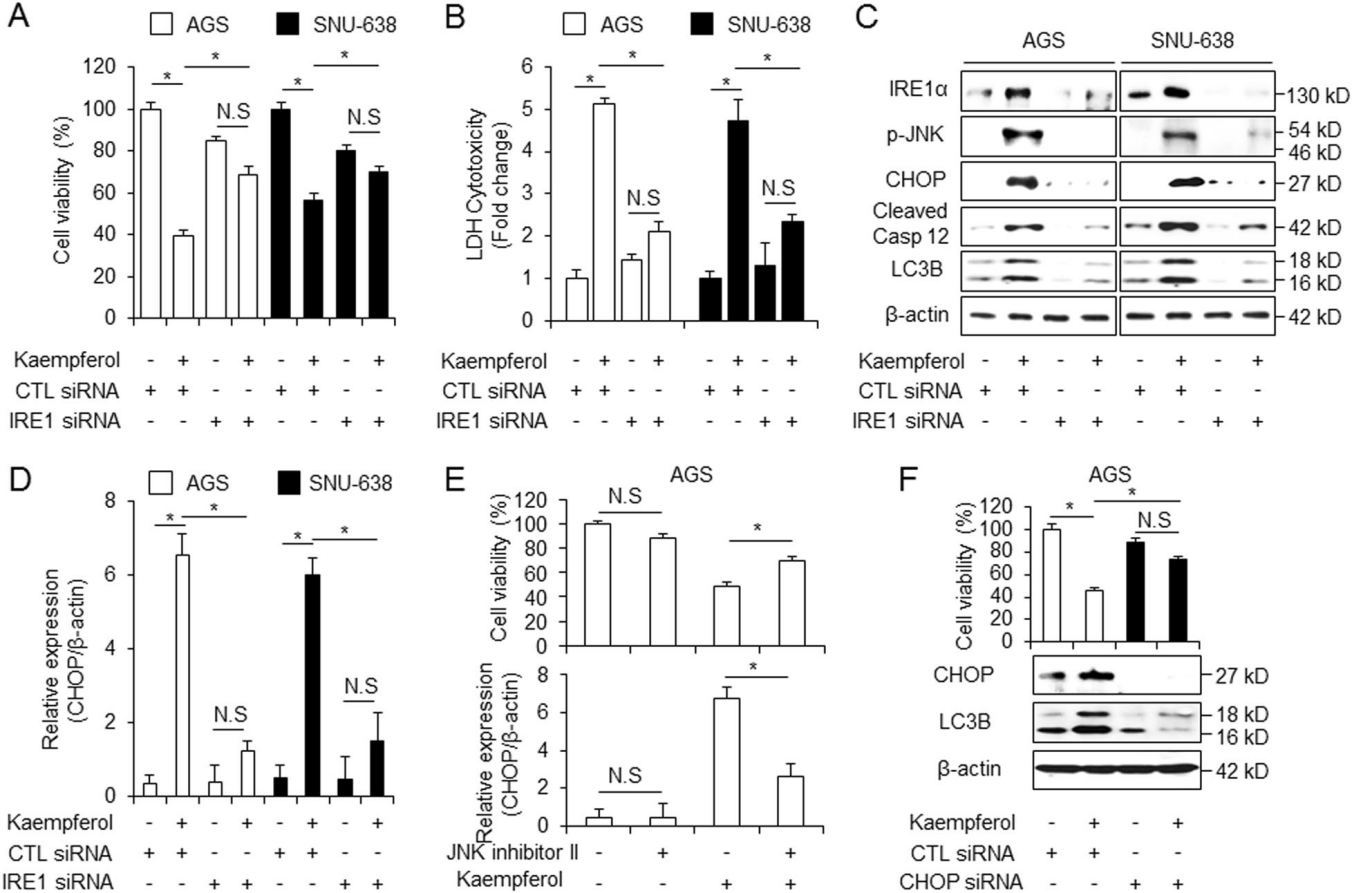
Inhibition of IRE1 pathway blocks cell death and autophagy in kaempferol-induced GC cells. **a**–**d** AGS and SNU-638 cells were transfected with IRE1 siRNA and treated with kaempferol. **a**, **b** Cell viability and LDH activity were determined by WST-1 and LDH assays, respectively; *p *<* 0.05. **c**, **d** IRE1α, p-JNK, CHOP, cleaved caspase-12, and LC3B were detected using western blot assay and the mRNA levels of CHOP were monitored using real-time RT-PCR. β-actin was used as a mRNA and protein loading control. **e** After AGS cells were pre-treated with JNK inhibitor II (20 μM, 24 h), they also were treated with kaempferol (50 μM, 24 h); *p *<* 0.05. Cell viability was determined using WST-1 assay, and mRNA levels of CHOP were assessed using real-time RT-PCR; *p *<* 0.05. **f** AGS cells were transfected with control or CHOP siRNA in the presence or absence of kaempferol. Cell viability was determined by the WST-1 assay and protein expression of CHOP and LC3B was detected using western blot assay. β-actin was used as a protein loading control.; *p *<* 0.05

Recent reports suggest that IRE1 signaling is the critical mediator of ER-stress-stimulated autophagy^42^. IRE1 is activated to transduce the ER stress signal to the cytosol (IRE1-JNK) to nucleus (CHOP) in cancer^43^. To confirm the role of IRE1 on kaempferol-induced ER stress, GC cells were transfected with IRE1 siRNA. Unlike in kaempferol-treated control siRNA-transfected cells, cell viability was significantly increased (Fig. 7a) and LDH release was lower (Fig. 7b) in kaempferol-treated IRE1 knockdown cells. Furthermore, the expression of IRE1, p-JNK, CHOP, cleaved-caspase-12, and LC3-II was more downregulated in kaempferol-mediated IRE1 knockdown cells than in kaempferol-treated control cells (Fig. 7c), and mRNA levels of CHOP were downregulated approximately 6-fold in kaempferol-treated IRE1-knockdown cells compare with kaempferol-treated control cells (Fig. 7d). We showed that kaempferol regulates JNK activation in AGS cells. Cell viability was increased and mRNA levels of CHOP were more downregulated to a greater extent by kaempferol+ JNK inhibitor treatment compared with kaempferol treatment alone (Fig. 7e), Furthermore, kaempferol increased cell viability and downregulated CHOP and LC3-II levels in CHOP knockdown cells to a greater extent than in control cells (Fig. 7f). This result indicates that IRE1 signaling knockdown inhibits kaempferol-treated autophagic cell death in GC.

### G9a inhibition regulates kaempferol-induced autophagic cell death in GC cells

**Fig. 8:**
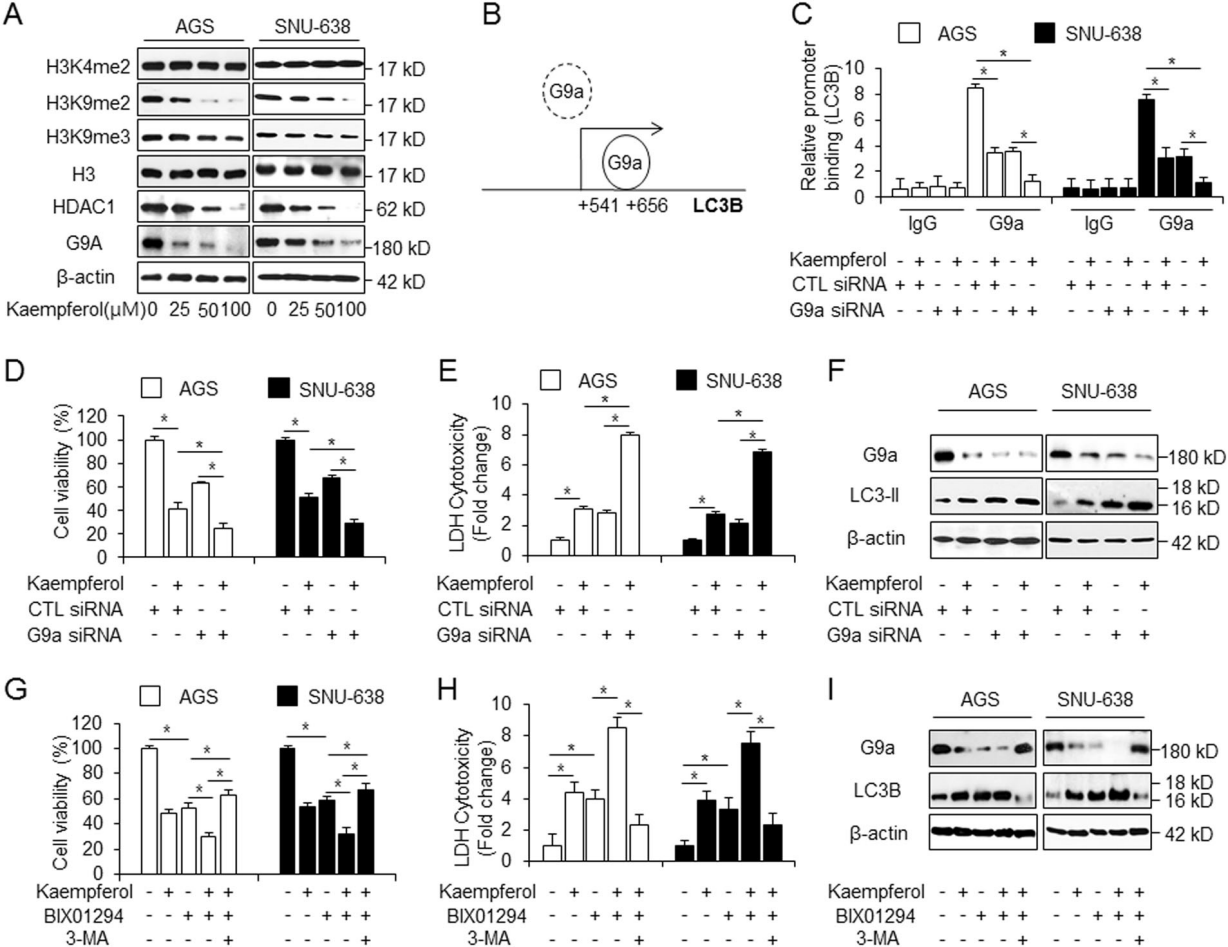
Inhibition of G9a regulates autophagic cell death in kaempferol-induced GC. **a** Protein levels of H3K4me2, H3K9me2, and H3K9me3 were investigated by western blotting in kaempferol-treated nuclear-fractioned AGS and SNU-638 cells, and H3 was used as a protein loading control. The protein level of G9a was detected by Western blot assay in kaemprefol-mediated AGS and SNU-638 cells and β-actin was used as a protein loading control. **b**–**f** AGS and SNU-638 cells were transfected with control or G9a siRNA based on the presence or absence of kaempferol. **b**, **c** The localization of the G9a on the *LC3B* promoter. Kaempferol regulates G9a binding at the *LC3B* promoter. Kaempferol (50 μM, 24 h) treatment was performed in control and G9a knockdown AGS and SNU-638 cells, and real-time ChIP assays of the *LC3B* promoter region (+541 to +656) were performed with G9a antibody. **d**, **e** Cell viability and LDH activity were determined by WST-1 and LDH assays, respectively; *p *<* 0.05. **f** Protein expression of G9a and LC3-II was detected using western blot assay. β-actin was used as a protein loading control. **g**–**i** AGS and SNU-638 cells were treated with kaempferol (50 μM, 24 h) and/or BIX-01294(10 μM, 24 h). Another group also indicated that AGS and SNU-638 cells were pretreated with 3-MA for 4 h and then treated with kaempferol/BIX-01294. Cell viability was determined by WST-1 assay and LDH release was determined by LDH assay. Protein levels of G9a and LC3B were detected using western blotting. β-actin was used as a protein loading control; *p *<* 0.05

Accumulating evidences suggest that epigenetic modification may regulate autophagy process and cell death^44,45^. Kaempferol is associated with epigenetic modification via inhibition of histone deacetylases (HDACs)^7^. Suberoyl hydroxamic acid (SAHA), a HDAC inhibitor, induces mTOR inactivation and autophagic cell death^46^. HDAC inhibitors mediate the downregulation of mTOR/AMPK signaling and activation of the ER stress response and autophagy^47^. Epigenetic modification including G9a silencing regulates mTOR/AMPK pathway in cancer, indicating autophagic cell death^48^. To study epigenetic regulation of kaempferol-induced autophagy, we tested if specific histone marks are involved with the epigenetic modification on kaempferol-treated GC. We screened the expression levels of H3K4me2, H3K9me2, and H3K9me3 in a dose-dependent manner in kaempferol-treated GC cells (Fig. 8a). Consequently, histone lysine9 dimethylation (H3K9me2) was downregulated in kaempferol-treated GC cells. G9a, a H3K9me2-specific methyltransferase, is involved in the transcriptional regulation of autophagy-related genes^49^. Kaempferol also downregulates the expression of G9a in a dose-dependent manner in GC cells (Fig. 8a). This result suggests that kaempferol mediates autophagy via the inhibition of G9a in GC. BIX-01294, a specific inhibitor of G9a, induced autophagic cell death in several tumor cell types^50^. To identify the candidate regulators such as G9a, on the *LC3B* gene, binding sites of G9a in the *LC3B* promoter region (+541 to +656) were identified (Fig. 8b)^51^. We performed quantitative chromatin immunoprecipitation (qChIP) to identify the G9a binding on the *LC3B* promoter in GC cells (Fig. 8c). Chromatin samples from control and G9a knockdown cells grown under kaempferol treatment were immunoprecipitated with G9a antibody (Fig. 8c). This experiment suggested that G9a was able to bind the *LC3B* promoter in control GC cells. However, kaempferol-treated or G9a knockdown cells showed decreased binding of G9a, and kaempferol-treated G9a knockdown cells showed further reduced binding of G9a. To confirm whether G9a regulates kaempferol-induced autophagic cell death, GC cells were transfected using G9a-specific siRNA, and kaempferol treatment was additionally performed. Cell viability was more decreased by kaempferol in G9a knockdown cells than in control siRNA-transfected cells that underwent kaempferol treatment (Fig. 8d). LDH release was markedly enhanced in kaempferol-induced G9a knockdown cells than in control siRNA-transfected cells that underwent kaempferol treatment (Fig. 8e). Compared with transfection experiments using control siRNA, Western blotting indicated that G9a knockdown cells show upregulated LC3-II expression, and additional kaempferol treatment further inhibits G9a level and accumulates LC3-II level (Fig. 8f). To further identify whether combined treatment with kaempferol and BIX-01294 regulates autophagy-induced cell death, we performed cell viability assay. Cell viability was significantly decreased by kaempferol or BIX-01294, as compared with DMSO, and kaempferol + BIX-01294 were more reduced cell viability in GC cells, compared with kaempferol or BIX-01294 alone (Fig. 8g). Interestingly, kaempferol + BIX-01294 dramatically increased cell viability to a greater extent than kaempferol or BIX-01294 alone in 3-MA-pretreated GC cells (Fig. 8g). Unlike DMSO-treated cells, LDH release increased in kaempferol and BIX-01294-treated GC cells, respectively and it was more increased in kaempferol + BIX-01294-treated GC cells, compared with kaempferol and BIX-01294 alone (Fig. 8h). However, 3-MA-treated LDH release was decreased in kaempferol + BIX-01294-treated GC cells (Fig. 8h). Western blotting indicated that kaempferol + BIX-01294 downregulated the G9a level and upregulated the LC3B-II level in GC cells (Fig. 8i). Taken together, our findings suggest that kaempferol/BIX-01294 further activate autophagic cell death via inhibition of G9a; however autophagy inhibition suppresses kaempferol + BIX-01294-mediated autophagic cell death via recovery of G9a. Therefore, these observations demonstrate that G9a inhibition is essential for kaempferol-induced autophagic cell death in GC cells, and that kaempferol in combination with BIX-01294 is an effective chemotherapeutic strategy that operates via autophagic cell death in GC cells.

## Discussion

Accumulating evidence suggests that kaempferol, a well-known flavonoid, is a potential HDAC inhibitor and an anti-cancer reagent against many cancers, including GC^52^. Epigenetically modified HDAC inhibitor induces autophagic cell death in cancer^53^. However, the molecular mechanisms underlying kaempferol-induced autophagic cell death remain unclear. In the present study, our data indicated that kaempferol induces autophagic cell death in GC, and to our knowledge, this is the first research to identify that kaempferol promotes cell death via the IRE1–JNK–CHOP pathway. Furthermore, kaempferol causes autophagic cell death via an epigenetic modification involving G9a inhibition. Autophagy inhibition including inhibitor and siRNA regulate autophagic flux in kaempferol-induced cells, and autophagy inhibition decreased cell death by increasing cell viability and reducing LDH activity in kaempferol-treated GC cells. These results indicate that kaempferol induces autophagic cell death, but autophagy inhibition plays a functional role for cell survival, implying kaempferol-induced autophagic cell death.

On the basis of our findings, we hypothesized that kaempferol promotes autophagic cell death via ER stress in GC. IRE1 may initiate cell death, and the accumulation of JNK and CHOP also induced cell death^54^. In this study, we screened two ER stress signaling pathways: PERK–eIF2α–CHOP and IRE1–JNK–CHOP pathway. When we monitored PERK pathway in kaempferol-treated GC cells, Western blotting did not detected phosphorylation of PERK and eIF2α, indicating inactivation of PERK signaling. However, it found upregulation of IRE1, p-IRE1, p-JNK, CHOP, and cleaved caspase-12, indicating the activation of IRE-mediated ER stress. CHOP is an important factor of ER stress-induced cell death via PERK and IRE1 signaling, and JNK also induces cell death via the induction of CHOP and C-jun^55,56^. IRE1 induces cell death via the activation of JNK and CHOP and the inhibition of Bcl-2^57^. JNK1 induces the phosphorylation of Bcl-2 and promotes autophagic cell death by disrupting the Bcl-2–Beclin-1 complex^58^. Interestingly, the inhibition of IRE1 and JNK, indicating the upstream signal of CHOP, reduces autophagic cell death via the downregulation of CHOP. These data demonstrated that kaempferol induces autophagic cell death via the IRE1–JNK–CHOP signaling in GC, whereas IRE1 knockdown blocks kaempferol-treated autophagic cell death.

Recent reports indicate that BIX-01294 reduces H3K9me2 through the inhibition of G9a and induces autophagic cell death in cancer^59^. Furthermore, G9a represses the expression of LC3B by directly binding with the promoter H3K9me2, indicating repressive mark, and the inhibition of G9a activates LC3B^60^. Our data indicated that kaempferol and BIX-01294 accumulate LC3-II and reduce cell viability via G9a inhibition, and a loss-of-function study using specific siRNA for G9a suggests that kaempferol reduces the binding of G9a on *LC3B* promoter in G9a knockdown cells. However, 3-MA significantly blocks LC3-II and cell death and recovers G9a in kaempferol/BIX-01294-treated GC cells. Therefore, our findings suggest that kaempferol induces autophagic cell death via the IRE1–JNK–CHOP pathway and the HDAC/G9a pathway in GC.

Taken together, these findings support that kaempferol induces autophagic cell death via IRE1–JNK1-mediated Bcl-2–Beclin-1 dissociation in GC cells. Moreover, kaempferol epigenetically mediates autophagic cell death via HDAC/G9a pathway. A deeper understanding of the molecular mechanism of kaempferol may contribute to useful cancer therapeutic approaches.

## Materials and methods

### Cell culture

The human GC cell lines (AGS, SNU-216, NCI-N87, SNU-638, and MKN-74) were purchased from the Korean Cell Line Bank (Cancer Research Center, Seoul National University, Seoul, Korea). Cells were cultured in RPMI1640 medium (Welgene) supplemented with 10% fetal bovine serum (JR Scientific) and 100 μg/mL antibiotics (100 U/ mL penicillin and 100 μg/ mL streptomycin, Welgene) in a 5% CO_2_ humidified incubator at 37 °C.

### Cell viability assay

The WST-1 assay was performed according to the manufacturer’s instructions (Roche, Mannheim, Germany) with 10 μL of WST-1 reagent was added to each well of a 96-well plate (1 × 10^4^ cell/well). After 1 h of incubation using CO_2_ incubator, the conversion of WST-1 reagent into chromogenic formazan was evaluated with a spectrophotometer (Molecular devices, USA). On day 1 after cell seeding, cells were treated with various doses of kaempferol (Sigma) (25, 50, and 100 µM) at various time points (8, 16, and 24 h). Autophagy inhibitor, 3-MA (Sigma, 5 mM), chloroquine (Sigma, 20 µM), compound C (Sigma, 2 µM) and SBI-0206965 (Sigma, 10 μM) were added sequentially to FBS-free medium for 24 h to inhibit autophagy. A pan-caspase inhibitor, Z-VAD-FMK (R&D Systems, 50 μM), was added to FBS-free medium for 24 h to inhibition of apoptosis. Cells were treated with an ER stress inducer, thapsigargin (Sigma, 3 μM, 24 h), along with FBS-free medium to activate ER stress and JNK inhibitor II (Calbiochem, 20 μM, 24 h) was added to inhibit JNK signaling. In addition, a G9a inhibitor, BIX-01294(Sigma, 10 μM, 24 h), was added to activate autophagy via G9a inhibition.

### LDH assay

AGS and SNU-638 cells (1 × 10^4^ cells/well) were seeded into a 96-well plate with growth medium. To determine the LDH (Thermo Scientific Pierce) activity in supernatants, 100 μL of Reaction mixture was added and incubated for 30 min in a dark room. The LDH activity was measured by the absorbance of the samples at 490 or 492 nm using ELISA reader.

### Transfection

AGS and SNU-638 cells (3 × 10^5^ cell/well) were transfected with double-stranded siRNAs (30 nmol/mL) of LC3B, ATG5, ULK1, IRE1 (Santacruz), and CHOP (Bioneer) in 6-well plate for 24 h by the Lipofectamine 2000 (Invitrogen) method according to the manufacturer’s protocol and were then recovered in RPMI1640 medium (Welgene) containing 5% fetal bovine serum (Gibco) and 100 μg/mL antibiotics (100 U/mL penicillin and 100 μg/mL streptomycin, Gibco) for 24 h. After recovering, viable cells were calculated by WST-1.

### Isolation of total RNA and Protein

Total RNA (approximately 50–100 mg) from GC cells (2 × 10^6^ cell/well) in 100 mm cell culture dish was prepared using Trizol according to the manufacturer’s protocols (invitrogen, Carlsbad, CA, USA). Protein cell lysates were collected in RIPA buffer containing a protease inhibitor cocktail (Sigma) on ice for 30 min and were passed through an 18-gauge needle and spin down. The supernatant was analyzed for protein content using the BCA method (Thermo scientific, Pierce BCA Protein Assay Kit, USA).

### Real-time PCR and western analysis

CHOP expression level was measured by real-time PCR using cDNA synthesized from 5 ug of total RNA and a reverse transcription kit (Promega, Madison, WI). Triplicate reactions were performed for each sample using an ABI Power SYBR green PCR Master Mix (Applied Biosystems) with CHOP-specific primers [5′-ATGAGGACCTGCAAGAGGTCC-3′ (sense) and 5′-TCCTCCTCAGTCAGCCAAGC-3′ (antisense)] on a Roche LightCycler 96 (Roche). RNA quantity was normalized to β-actin primers [5′-AAGGCCAAC CGCGAGAAGAT-3′ (sense) and 5′-TGATGACCTGGCCGTCAGG-3′ (antisense)]. Gene expression was quantified according to the 2^-ΔCt^ method. To conduct the Western blot assay, GC cell lines were solubilized in radioimmunoprecipitation assay (RIPA) lysis buffer [50 mM/L Tris-HCl (pH 7.4), 150 mM/L NaCl, 1% NP40, 0.25% sodium deoxycholate, 1 mM/L phenylmethylsulfonylfluoride (PMSF), 1 mM/L sodium orthovanadate, and 1 × sigma protease inhibitor cocktail] and protein content was measured using a standard bicinchoninic acid assay. Equal amounts of protein (20 μg) were size-fractionated by 8–15% SDS-PAGE and then transferred onto an NC membrane (Millipore Corporation, Billerica, MA, USA). Membranes were blocked by incubation for 30 min with 5% skim milk/PBS-T [PBS with 5% powdered milk (BD) and 1% Tween20 (Sigma)], and incubated overnight at 4 °C with primary antibodies diluted in 1 × PBST buffer. The following primary antibodies were used: β-actin, Bcl-2, Beclin-1, ULK1, eIF2α, JNK, and ATG5 (Santa Cruz, 1:1000), LC3B (Sigma, 1:1000), p62 (Sigma, 1:1000), p-IRE1 (Abcam, 1:1000), and G9a (Abcam, 1:1000), cleaved caspase-3, cleaved caspase-9, cleaved PARP, p62, p-AMPKα (Thr172), AMPKα, p-mTOR (Ser2448), mTOR, p-ULK1 (Ser555), p-P70S6K (Thr389), P70S6K, IRE1, PERK, p-PERK (Thr980), p-eIF2α (Ser51), p-JNK, H3, H3K4me2, H3K9me2, H3K9me3, and CHOP (CellSignaling, 1:1000). The membranes were washed three times with PBST buffer. A secondary antibody diluted in PBST or TBST buffer was added, and incubation was done for 40 min at room temperature. The following secondary antibodies were used: anti-rabbit IgG HRP-linked antibody and anti-mouse IgG HRP-linked antibody (KPL, 1:6000). The membranes were washed six times with PBST buffer for 1 h. The blots were visualized using Western chemiluminescent HRP substrate (Millipore).

### Quantification of pEGFP-LC3 puncta

AGS and SNU-638 cells (2 × 10^5^ cells per well) in a 6-well plate were transfected with pEGFP-LC3 using lipofectamin 2000 (Invitrogen), and then treated with 50 μM kaempferol for 8 h. A pEGFP-LC3B-positive punctate pattern was observed by confocal microscopy. Confocal microscopy was conducted using a ZEISS LSM5 PASCAL confocal microscope with 405- and 488-nm excitation lasers.

### Nuclear fraction

Nuclear fraction was carried out using Nuclear Extract kit (Active motif) according to manufacturer’s instructions. After the cytoplasmic fraction was extracted as supernatants, cell pellets were resuspended in 50 ml of complete lysis buffer and supernatants were used as nuclear fractions by centrifugation at 14,000 × *g* for 10 min at 4 °C.

### Immunoprecipitation (IP) assay

We extracted cell lysates from AGS cells (2 × 10^6^ per well) on 100 mm cell culture plate in a IP buffer (pH 7.5) containing 50 mM Tris-HCl, 250 mM NaCl, 5 mM EDTA, 0.5%(v/v) NP-40, and protease inhibitor cocktail (Sigma). We incubated anti-Bcl-2 (Santa Cruz) and anti-BECN-1 (Santa Cruz) with lysate at 4 °C for 16 h. We used protein A/G PLUS agarose (Santa Cruz) to pull down immunocomplexes. We washed precipitates three times with IP buffer. We resolved the immunoprecipitated proteins by 12% SDS-PAGE and analyzed them.

### Chromatin immunoprecipitation(ChIP) assay

ChIP assays were performed using an EZ ChIP Chromatin Immunoprecipitation kit (Millipore, Billerica, MA, USA) as described in the supplier’s protocol. Briefly, the cross-linked chromatin was sonicated after cell lysis and then incubated overnight at 4 °C with antibodies against G9a (Abcam). The immunocomplex was precipitated with protein A–agarose (Millipore), and the beads were washed, sequentially treated with 10 µl of RNase A (37 °C for 30 min) and 75 µl of proteinase K (45 °C for 4 h), and incubated at 65 °C overnight to reverse cross-link the chromatin. The DNA was recovered by phenol–chloroform extraction and co-precipitation with glycogen and was then dissolved in 50 µl of Tris-EDTA (TE) buffer. DNA associated with the ER was amplified by PCR using 1 µl of precipitated DNA. PCR primers [5′-GAAGTGGCTATCGCCAGAGT-3′ (sense) and 5′- GCTGCTTGAAGGTCTTCTCC -3′ (antisense)] were designed to amplify the G9a binding site at the LC3B gene promoter. Quantitative PCR conditions were 40 cycles at 94 °C for 40 s, 60 °C for 1 min, and 72 °C for 40 s.

### Statistical Analysis

All results were confirmed in at least three independent experiments; Student’s *t*-tests were used for between-groups comparisons of the means of quantitative data, and *p* *<* 0.05 was considered statistically significant.
